# The Influence of Perceptual-Motor Variability on the Perception of Action Boundaries for Reaching in a Real-World Setting

**DOI:** 10.1177/03010066211038406

**Published:** 2021-08-23

**Authors:** Lisa P. Y. Lin, Christopher J. Plack, Sally A. Linkenauger

**Affiliations:** Department of Psychology, 4396 Lancaster University, Lancaster, UK; Department of Psychology, 4396 Lancaster University, Lancaster, UK; Manchester Centre for Audiology and Deafness, 5292 University of Manchester, Manchester, UK; Department of Psychology, 4396 Lancaster University, Lancaster, UK

**Keywords:** perception/action, perceptual learning, reaching/grasping, perceptual-motor calibration, action boundaries

## Abstract

The ability to accurately perceive the extent over which one can act is requisite for the successful execution of visually guided actions. Yet, like other outcomes of perceptual-motor experience, our perceived action boundaries are not stagnant, but in constant flux. Hence, the perceptual systems must account for variability in one’s action capabilities in order for the perceiver to determine when they are capable of successfully performing an action. Recent work has found that, after reaching with a virtual arm that varied between short and long each time they reach, individuals determined their perceived action boundaries using the most liberal reaching experience. However, these studies were conducted in virtual reality, and the perceptual systems may handle variability differently in a real-world setting. To test this hypothesis, we created a modified orthopedic elbow brace that mimics injury in the upper limb by restricting elbow extension via remote control. Participants were asked to make reachability judgments after training in which the maximum extent of their reaching ability was either unconstricted, constricted or variable over several calibration trials. Findings from the current study did not conform to those in virtual reality; participants were more conservative with their reachability estimates after experiencing variability in a real-world setting.

Successful performance of action relies on the accurate perception of opportunities for action in the environment; in particular, action boundaries. Action boundaries are learned over time and are the limitations of a perceiver’s action capabilities. They distinguish between possible and impossible actions, and action is only possible if it is within the perceiver’s action boundary (Fajen, 2005). For instance, when intending to reach, the length and flexibility/range of movement (ROM) of an individual’s arm determine the maximum extent of their reachability. For an object to be reachable, the distance to the target must be within the perceiver’s action boundary for reaching. The ability to accurately perceive one’s action boundaries in relation to the environment is requisite for the successful execution of actions.

An ample body of research has demonstrated that people could readily perceive their action boundary for different types of actions and across different environmental contexts. For example, individuals are remarkably accurate at judging the maximum height of steps that they can climb, and irrespective of body height, individuals judge the maximum climbable stair height as a constant proportion of leg length (Warren, 1984). Other studies have demonstrated people’s sensitivity to their action boundaries for a variety of actions, including, but not limited to, passing through doorways (Franchak & Adolph, 2012; Warren & Whang, 1987), fitting hand through apertures (Ishak et al., 2014), grasping (Linkenauger et al., 2011), and reaching (Carello et al., 1989; Linkenauger et al., 2009). Furthermore, individuals are able to recalibrate to new action boundaries following changes in their action capabilities and/or environmental constraints. Such examples include calibrating their maximum sitting and stepping height judgments while wearing blocks under their feet (Hirose & Nishio, 2001; Mark, 1987), adjusting their judgment of passability when fitting one’s hand through an opening with a prosthesis attached to their hand (Ishak et al., 2008), and updating their reachability judgment when their arm’s reach has been extended or constricted in a virtual environment (Linkenauger et al., 2015).

However, like other outcomes of perceptual-motor experience, our perceived action boundaries are not immutable, but in constant flux. These changes take place over different timescales and have consequences for actions. Long-term changes take place via naturally occurring changes in physiology and perceptual-motor capabilities associated with growth and aging (Comalli et al., 2013; Konczak et al., 1992), whereas short term changes in action boundaries, such as injuries, fatigue and posture, could occur at any time and bring about inconsistent fluctuations in the perceptual-motor feedback specifying one’s action boundaries, which has consequences for motor performance (Franchak & Adolph, 2014). In addition to these changes, it is not possible for one to execute actions with perfect consistency. Thus, regardless of how consistent an action’s outcome may appear, some degree of variability is always present in the perceptual-motor information upon which our perception of action boundaries is based. Consequently, when determining one’s perceived action boundary, the perceptual-motor system must account for variability in perceptual-motor feedback in order to perform actions adaptively and minimize performance errors. How does the perceptual system determine the maximum extent over which an action can be performed when the information that we based our perception of this extent on is inconsistent?

One possible method the perceptual system could employ would be to use the average experienced reach to generate the most statistically likely outcome (Deneve & Pouget, 2004; Kording & Wolpert, 2006). Take, for example, an observer who has experienced two different sized action boundaries (large and small) with equal probability during their reaching experience. If they use the average of their reaching experience to determine the action boundary, the selected action boundary should be identical to the mean, as the two action boundaries experienced were of equal probability. Conversely, if the smaller action boundary was experienced significantly more often than the larger, then we would expect a shift towards the smaller action boundary as it would be more statistically likely than the larger action boundary.

Although this approach may allow the perceiver to determine perceived action boundaries under conditions of uncertainty in a more optimizing manner, it would also be a more time consuming and energetically costly approach due to the amount of information processing involved (Howarth et al., 2010). Furthermore, both human cognitive and bioenergetic resources are limited (Niven & Laughlin, 2008), and not every action execution nor circumstance is important enough to justify expending resources to integrate probabilistic information and/or to formulate optimal solutions. Therefore, as another approach, the perceptual system could use heuristics as an effort-reduction strategy, or when the cost of information processing outweighs potential gain in judgment accuracy. It should be noted that the current study was framed as a test between optimizing versus satisfying approaches, and our hypothesis was not based on ecological approaches of visual perception, but rather evolutionary approaches. Hence, one could consider fewer alternatives by disregarding probabilistic information to make decisions that are just “good enough” (Gigerenzer & Gaissmaier, 2011; Hogarth & Karelaia, 2007). One such heuristic would be to select the action boundary using the most liberal reach experienced; this method would maximize the number of successful attempts, but at the same time it may also result in the highest number of unsuccessful attempts. Thus, this approach would only be appropriate in the absence of negative consequences associated with failed action. Another possible heuristic would be to select an action boundary using the most conservative sized reach experienced, such as in particular in situations where the penalties for selecting the inappropriate action boundary are high. This approach would result in the smallest number of successful attempts, but also the smallest number of failed attempts. Alternatively, the perceptual system could select a moderate-sized action boundary; selecting an action boundary size that is in between the most liberal and most conservative action boundary would allow the perceptual system to balance the number of successful attempts with the number of unsuccessful attempts. Taken together, it is reasonable to postulate that, to maximize efficiency, the perceptual system would utilize different strategies on an ad hoc basis to determine perceived action boundaries under conditions of uncertainty.

Recent research has investigated participants’ judgment of action boundaries for reaching following changes in their action capabilities in virtual environments. Lin et al. (2020) had participants estimate their action boundary for horizontal reaching following calibration to a long virtual arm (extended reach condition), a short virtual arm (constricted reach condition), or a virtual arm that varied in size randomly between a long virtual arm, medium virtual arm, and short virtual arm each time they reached. They found that individuals were able to calibrate to changes in their action capabilities and their selected action boundaries were consistent with their reaching experience during calibration. They estimated their reachability to be significantly farther in the extended reach condition than in the constricted reach condition. However, in the variable condition in which they experienced three arm’s reaches with equal probability, individuals tended to indicate that their perceived action boundary for reaching more resembled their experience with the longer reaches than with the shorter reaches. Had they used the averaged reaching experience to determine their perceived action boundary, the difference between extended and variable conditions would be similar to the difference between constricted and variable conditions. Instead, they found that the difference between the extended and variable conditions was significantly smaller than the difference between the variable and constricted conditions, indicating that the estimates in the variable reach condition were closer to the extended reach estimates than the constricted reach estimates, and individuals in the variable reach condition had estimated liberally rather than conservatively. These findings suggest that the perceptual system employs a liberal tactic rather than an average to determine perceived action boundaries for reaching in the event of perceptual-motor variability.

Actions cannot be performed the same way repeatedly and variability in the outcome is always present, but the link between variability and perceptual estimates is often ignored in affordance literature. This set of studies has provided insights into the possible mechanism by which the perceptual system accounts for perceptual-motor variability when determining perceived action boundaries, and they have exposed a gap in the literature that is important to fill if we are able to fully understand the nature of affordance perception. However, these studies were conducted in a virtual environment and the extent to which these findings are generalizable to the real world is as yet unknown. In the real world, some research has demonstrated that people are sensitive to their movement variability and take their task-relevant movement variability into account when making action boundary judgments for actions such as aperture passing (Hackney & Cinelli, 2011; Lucaites et al., 2020; Wilmut & Barnett, 2010, 2011; Wilmut et al., 2015) and stepping over obstacles (Snapp-Childs & Bingham, 2009). However, only individual variability in natural postural sway and stability/motor control during movement were considered. As mentioned above, not all perceptual-motor variability is large enough to be detectable when learning action boundaries, but in some instances, perceptual-motor variability is quite evident. Hence, to assess how the perceptual system accounts for overt perceptual-motor variability in motor experience and recalibrate to new action boundary following changes in action capabilities in the real world, it would be desirable to use large and observable changes in arm’s reach during the perceptual-motor calibration/experience.

In Lin et al. (2020), reaching ability was manipulated by modifying the length of the virtual arms by 50% more or less than the participant’s actual arm length. Yet, similar changes in arm length would be difficult or nearly impossible to accomplish in the real world due to the constancy of the body morphology. Therefore, in the present study, we opt to manipulate reaching ability by restricting the range of motion of the elbow using a modified orthopedic elbow brace. In addition to its use in rehabilitation treatments, previous research has used a similar device to identify the necessary functional range of motion of the elbow for everyday activities (e.g., Vasen et al., 1995). Elbow mobility is essential for upper limb function; a 50% reduction of elbow motion represents ∼80% loss of upper limb function (Fusaro et al., 2014). Stiffness of the elbow is a common occurrence after injury and can be defined as a loss of extension >30° and/or flexion of <120° (Sojbjerg, 1996), and the loss of elbow extension is more frequently encountered than flexion loss (Charalambous & Morrey, 2012). Hence, it is a debilitating condition that has detrimental consequences for the individual’s ability to perform daily activities (Bartoszek et al., 2015). By isolating the allowable range of motion of the elbow, we would be able to simulate the movement of the arm in a state of injury and introduce variability into one’s perceptual-motor feedback for reaching in a controlled manner while still in the real world.

The present study aimed to use the elbow brace to establish whether the perceptual system utilizes the same strategy in a real-world situation as in virtual environments. Participants were asked to make reaching ability judgments after training that the maximum extent of their reaching ability is either constricted (limited to 60° extension), unconstricted (0° extension), or variable (varied randomly between 0°, 30°, and 60° extension). Our manipulation was intentionally large to create a detectable difference in the dependent measure across the different conditions. In light of the findings from Lin et al. (2020), and given the context and task similarity, we expected participants to remain relatively liberal with their reachability estimates as they did in a virtual environment, but to a lesser degree. The latter is because, while the action they had to perform was the same (i.e., reaching horizontally), the changes in their reaching ability were less drastic. Additionally, the changes in their reaching were employed in a different manner. In the virtual environment, arm length was modified, and range of motion was intact; whereas, here, we limited range of motion while arm length remained intact. Thus, it is possible that limiting elbow range of motion mimics the movement of the arm in a state of injury, and the perceptual system would treat the restricted movement of the arm as if it were a real injury, which could induce participants to be more conservative to prevent further injury.

## Method

### Participants

G*Power software application (Faul et al., 2007) was used to perform an a priori power analysis to estimate sample sizes required to achieve adequate power. The required power was set at 1−*β* = 0.85, and the level of significance was kept at *α* = 0.05. We expected a medium effect size of 0.25 due to the novelty of the paradigm. Power analysis indicated that a sample of *N* = 8 would be sufficient to achieve a power of 0.85 and an alpha of 0.05. We have doubled the number and increased our sample size to 16 participants.

Sixteen participants (four males) between 18 and 21 years of age (*M*_age_ = 19.13, *SD_age_* = .89) were recruited from Lancaster University through opportunity sampling. All participants, but three, were reported to be right-handed. All participants had normal or corrected-to-normal vision. All participants provided informed consent. The study was approved by the Faculty of Science and Technology Research Ethics Committee at Lancaster University.

### Stimulus and Apparatus

The participant sat in front of a rectangular table onto which stimuli were projected from a projector mounted on the ceiling. The table (120 cm × 80 cm × 71 cm) was covered with a piece of black cardboard (56 cm × 82 cm) to create a uniform background and minimize landmarks that could influence participants’ judgments. A white dot (2 cm in diameter) was projected on the edge of the black cardboard directly in front of the center of the participant’s body from the projector. This dot was used to represent the center of the participant’s body and was used as a consistent reference point for measuring reachability during the estimation phase.

**Figure 1.**
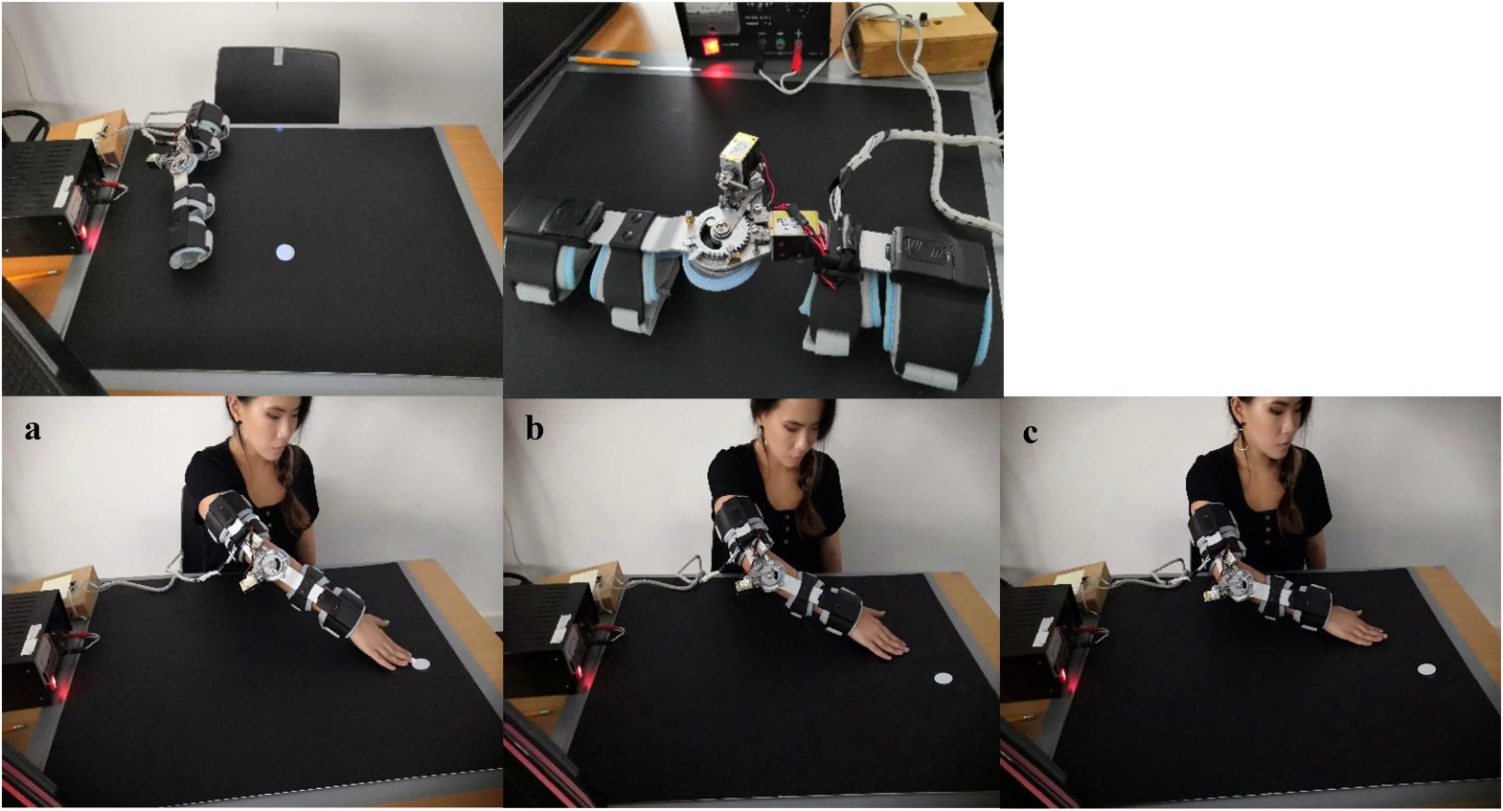
Top panel: Experimental set-up and apparatus. Bottom panel: Illustration of a participant completing a calibration trial with either a long reach (a), medium reach (b), or short reach (c).

Reachability was manipulated by using a device that was made of a modified orthopedic elbow brace with adjustable ROM. The modified elbow brace was 42 cm in length and weighed 0.6 kg. Two electric mini motors were added to the rotation hinge of the elbow brace to allow systematic manipulation of the ROM by restricting the extension of the elbow. Three different reaches were used, and the amount of extension of the elbow was limited in 30° increments. For the long reach, the ROM of the elbow was not limited (0° of extension); for the medium reach, elbow extension was limited to 30°; and for the short reach, elbow extension was limited to 60° (see Figure 1).

### Procedure

After providing informed consent, participants were asked to sit between the table and the wall, in that their body was touching the table and their core was aligned with the reference point projected onto the table. They were given instructions for both the calibration and estimation phases of the experiment. After donning the elbow brace on their right arm, participants completed all three experimental conditions, and participants were randomly assigned to different orders of conditions. In the *unconstricted* reach condition, despite wearing the elbow brace on their arm, participants’ elbow ROM was completely unconstricted (0° extension). In the *constricted* reach condition, participants’ elbow extension was limited to 60°. In the *variable* reach condition, participants’ elbow ROM varied between the long reach (0° extension), the medium reach (30° extension), and the short reach (60° extension). In the variable condition, reaches changed randomly in between each calibration trial and participants experienced all three reaches with equal probability (i.e., an equal number of trials); all reaches were experienced in a randomized order.

**Figure 2.**
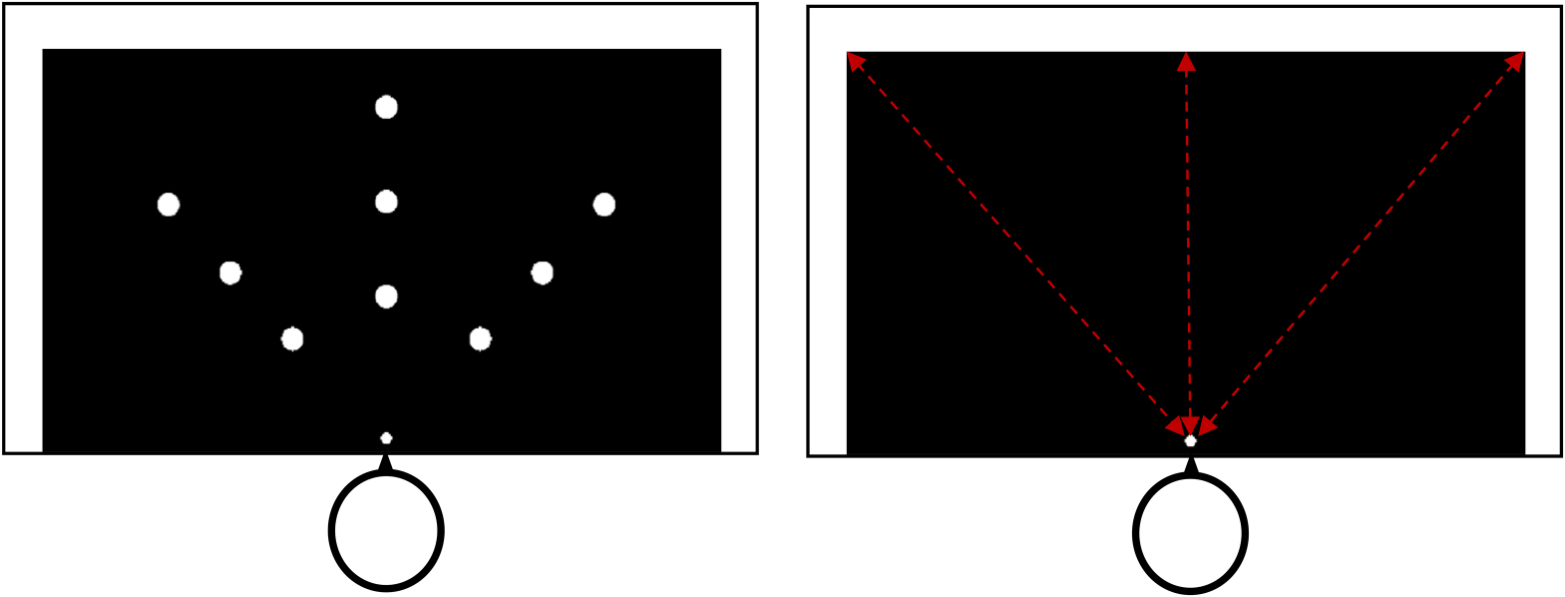
Left panel: Diagram of the calibration phase. The large white dots represent the nine possible dot locations and the smaller white dot represents the reference dot. Right panel: Diagram of the estimation phase. The red dotted lines represent the axis upon which the red laser pointer moved.

Each condition consisted of two phases: calibration and estimation. The calibration phase consisted of 36 trials in which a white dot (4 cm diameter) was presented on the left, right or in front of the participant. Participants were instructed to reach and touch the white dot with their hand. If the dot was too far for them to reach, they were instructed to point towards it instead. After they reached out and touched/pointed the dot, the dot disappeared and another white dot at a different location appeared. The dots were presented at one of the three horizontal distances from the reference point (20 cm, 35 cm, 50 cm) and the dots were either presented directly in front of the participants or 15, 25, or 35 cm to the left or the right of the central line, for a total of nine possible dot locations. Each location was presented four times in a random order for a total of 36 trials. Participants engaged in an estimation phase after each calibration phase. The estimation phase consisted of 12 trials, in which participants reported their maximal reaching ability by instructing the experimenter to move the estimation dot (using a laser pointer) closer or farther until it was at the maximum distance the participant believes that they could reach. During the estimation phase of all reaching conditions, the elbow brace remained on the participants’ right arm, but they were instructed to place their arms underneath the table so that they had no visual feedback of their arm’s location. To control for hysteresis, in half of the trials, the estimation dot’s starting position was directly in front of the participant and at the reference point; participants moved the dot away from them in one of three directions: contralateral, straight, and ipsilateral (near left, near center, near right). For the other half of the trials, the estimation dot’s starting position was at the central edge of the black cardboard or 41 cm to the left or the right (far center, far left, far right); these dots moved straight or diagonally towards the reference point. The dots either started close to or far away from the participants and were presented directly in front of or to the left or right, for a total of six locations (near/far left, near/far center, near/far right) each presented twice for a total of 12 trials. Participants were encouraged to make as many adjustments as necessary for an accurate estimation of their reachability and to move the estimation dot beyond the black cardboard if they thought it was necessary, then close their eyes in after each trial while the experimenter measured the distance between the reference point and the final location of the estimation dot landed. Figure 2 illustrates the dot locations for the calibration and estimation phases.

## Results

Estimated reachability was defined as the farthest extent to which participants estimated they could reach. To analyze the influence of reaching condition on estimated reachability, we employed a repeated measures one-way analysis of variance (ANOVA) with reaching condition (unconstricted/ constricted/variable) and direction (left/right/center) as within-subject factors and estimated reachability as the dependent variable.

**Figure 3.**
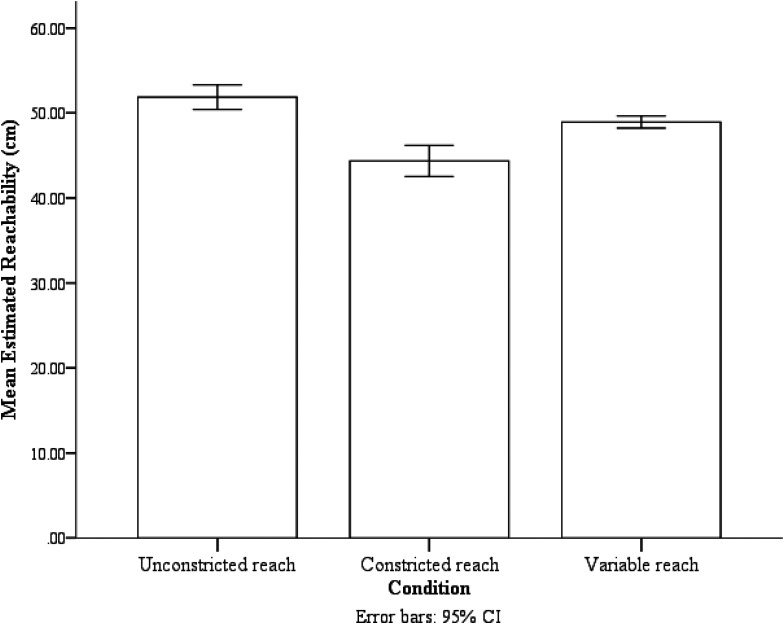
The mean estimated reachability of the three reaching conditions. Error bars are 95% confidence interval (CI) calculated within-subject with the method provided by Loftus and Masson (1994).

The analysis provided Greenhouse-Geisser corrected degrees of freedom to account for possible violations of sphericity, therefore the degrees of freedom were not always integers. Analysis showed effects of reaching condition on estimated reachability, *F*(1.19, 17.90) = 21.84, *p* < .001, *ƞ_p_*^2^ = 0.59. Bonferroni-corrected post hoc analysis (*t*-test) showed that participants estimated the extent of their reach as being farther in the unconstricted reach condition (*M* = 51.86 cm, *SE* = .84 cm) than in the constricted reach condition (*M* = 44.35 cm, *SE* = 1.91 cm, *p* = .001). They also estimated their reachability to be farther in the variable reach condition (*M* = 48.93 cm, *SE* = 1.16 cm, *p* = .003) than in the constricted reach condition. Furthermore, they estimated their reachability to be farther in the unconstricted reach condition than in the variable reach condition (*p* = .001), see Figure 3.

**Figure 4.**
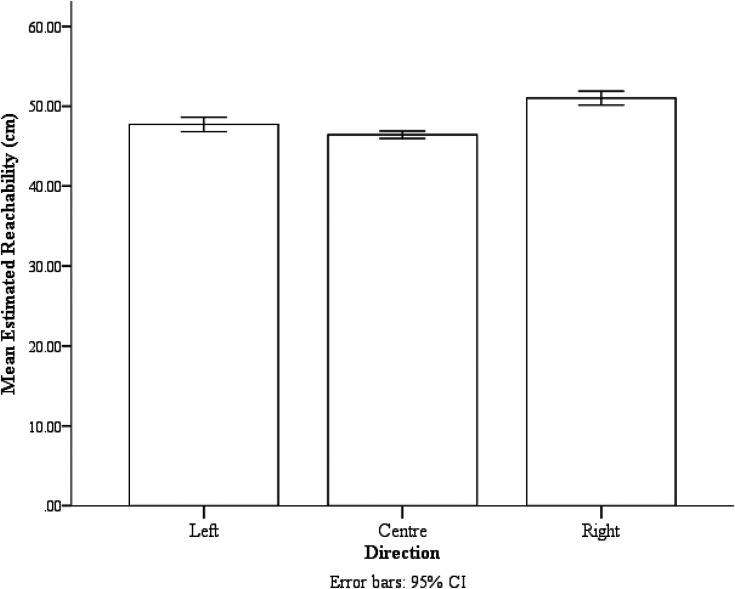
The mean estimated reachability of the three reaching directions. Error bars are 95% confidence interval (CI) calculated within-subject with the method provided by Loftus and Masson (1994).

The direction also significantly influenced estimated reachability, *F*(1.41,21.19) = 28.06, *p* < .001, *ƞ_p_*^2^ = 0.65. Participants estimated their reachability for targets on the right (*M* = 51.01 cm, *SE* = 1.40 cm) to be farther than targets on the left (*M* = 47.72 cm, *SE* = 1.21 cm, *p* = .003) and farther than those in the center (*M* = 46.42 cm, *SE* = 1.17 cm, *p* < .001). The evidence was inconclusive for the estimated reachability of targets on the left and in the center, *p* = .10 (see Figure 4).

**Figure 5.**
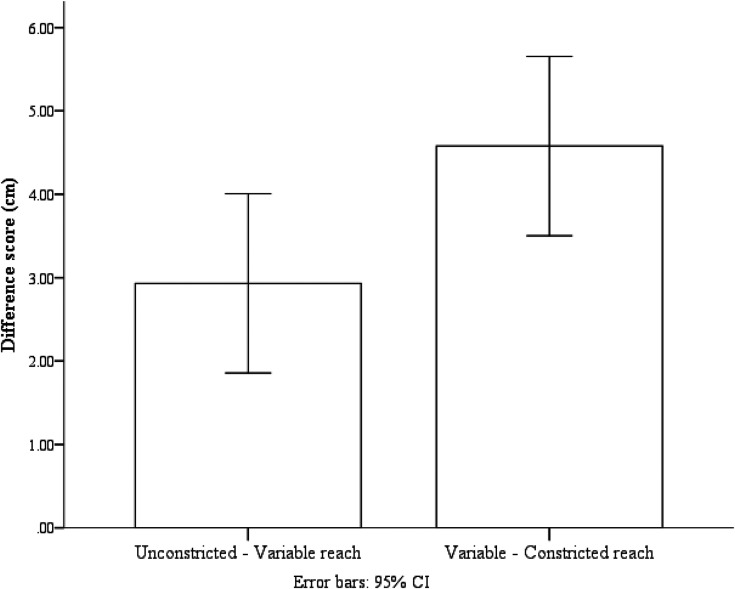
The UV and VC difference scores. Error bars are 95% confidence interval (CI) calculated within-subject with the method provided by Loftus and Masson (1994).

To get a better idea of the relations between the three conditions, for each participant in each condition, we created two scores. We created one difference score by subtracting the mean variable reach estimate from the mean unconstricted reach estimate (UV), and the other difference score was created by subtracting the mean constricted reach estimate from the mean variable reach estimate (VC). If participants used the average experienced reach to determine their action boundaries, we should expect no difference between the UV and VC scores. A paired-sample *t*-test was conducted to compare the difference between the UV and VC scores. The *t*-test found no evidence for a difference between the UV scores (*M* = 2.93 cm, *SD* = 2.60 cm) and the VC scores (*M* = 4.58 cm, *SD* = 4.44 cm); *t*(15) = −1.63, *p* = .12 (see Figure 5). These findings indicate that, after experiencing random variability in their reaching experience, participants were more conservative with their reachability estimates than those reported in previous studies conducted in virtual reality, and participants selected a moderate size action boundary that was in between the unconstricted reach condition and the constricted reach condition.

One possibility is that the reaching experience from prior conditions could influence the reachability estimates in the latter conditions, and we do not doubt that some influence across the conditions occurs as with any form of perceptual-motor learning. Although we fully counterbalanced across participants, which would have eliminated/minimized any systematic bias in the conditions as a result of the order, we felt it is important to assess if there was any cumulative effect from reaching experience in prior conditions that influenced estimates in the subsequent conditions.

**Figure 6.**
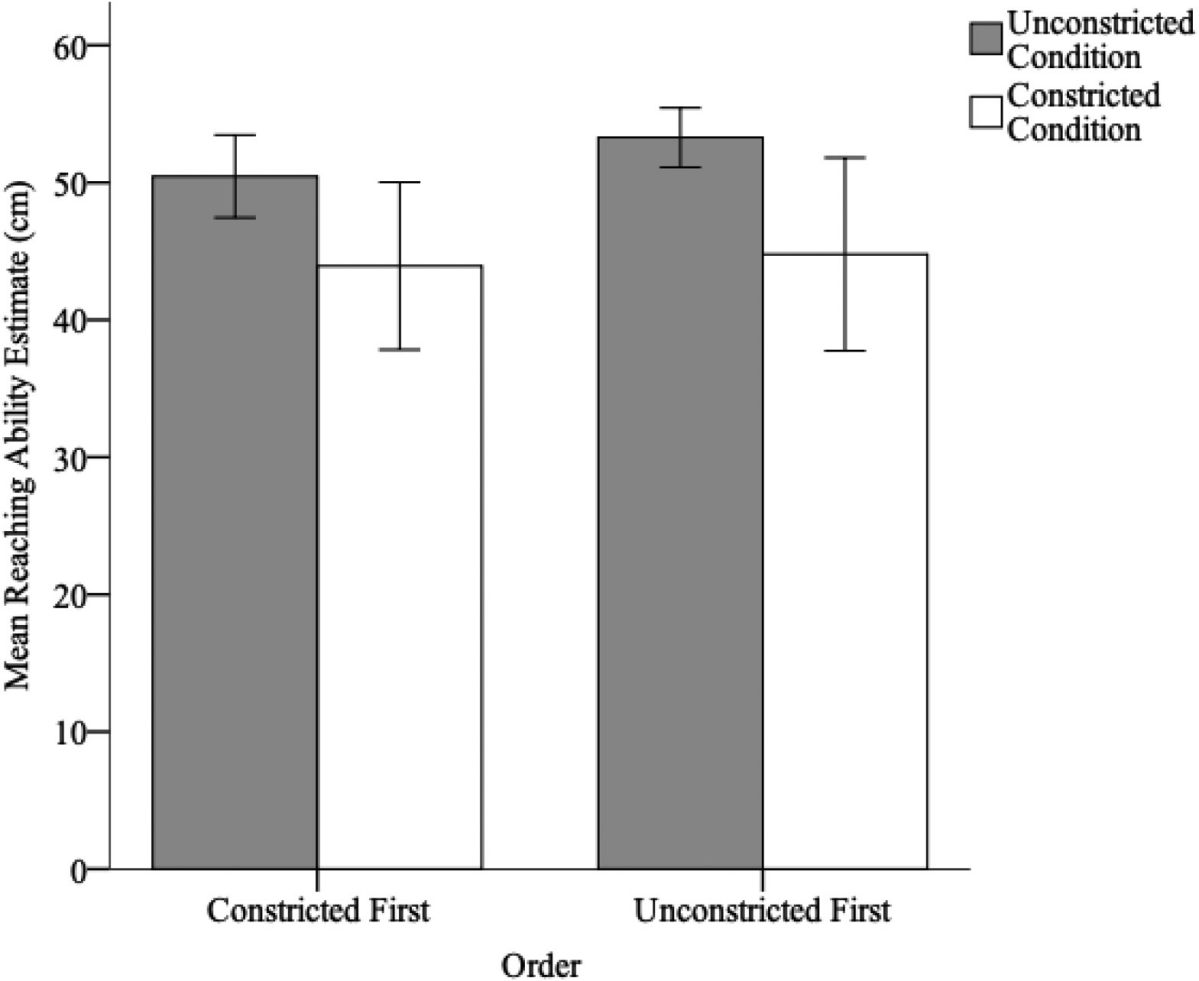
Reaching ability estimates for the constricted and unconstricted conditions for those who completed the constricted versus the unconstricted condition first. Error bars represent 95% confidence intervals (CIs).

Hence, we created an order variable in which we dummy coded participants who engaged in the unconstricted reach condition prior to the constricted reach condition as 1 and those who engaged in the constricted reach condition prior to the unconstricted reach condition as 2. We expected that if the prior condition had any meaningful influence on the subsequent condition, then estimates in the constricted reach condition would be larger if it was conducted *after* the unconstricted reach condition, and the estimates in the unconstricted reach condition would be smaller if it was preceded by the constricted reach condition. To assess this possibility, we conducted a repeated-measures ANOVA, with condition (constricted vs. unconstricted) as a within-subject factor and order as a between-subject factor. If there were order effects, then both constricted and unconstricted reach estimates should be higher in order 1 than in order 2. Conversely, if there were not order effects, then both constricted and unconstricted reach estimates in order 1 should be similar to those in order 2. As expected, we found a significant effect of condition, *F*(1, 14) = 23.72, *p* < .001, with unconstricted reach estimates being larger, *M* = 51.86 cm, *SE* = 0.79 cm, than constricted reach estimates, *M* = 44.35 cm, *SE* = 1.97 cm. We found no effect of order, *p* = .49, or a significant interaction between order and condition, *p* = .53 (see Figure 6).

To investigate this in more detail, we conducted two between-subjects *t*-tests to independently assess the effects of order on the constricted reach condition and on the unconstricted reach condition. We found no significant effect of an order for either condition (*p* = .83 and *p* = .10, respectively). These negative results could be due to a lack of power. However, the analyses do confirm that the reaching experience within each condition had a large effect on the reachability estimates; whereas, the reaching experience in each condition likely had a small/negligible influence on the other conditions.

## Discussion

In the current study, we examined the effect of random perceptual-motor variability on action boundary perception for reaching in a real-world setting. We manipulated participants’ reaching capabilities by restricting the degree to which they could extend their elbow. Participants were asked to make reachability judgments after training that the maximum extent of their reaching ability is either unconstricted (0°extension), constricted (60° extension) or variable (elbow ROM varied between 0°/30°/60° extension).

We found that the perceived action boundary for reaching significantly varied with respect to reaching calibration conditions. The relative difference between conditions was similar to previous studies conducted in virtual reality, suggesting that in addition to artificial body extension and tool use, perceived reachability can also be manipulated by changes in elbow ROM and that a large degree of *controlled* perceptual-motor variability can be introduced into one’s perceptual-motor feedback associated with motor learning in the real world.

Although participants continued to show a slight trend towards liberal estimates in the variable condition as in the virtual reality studies of Lin et al. (2020), this effect was not significant. In Lin et al. (2020), the difference between the extended reach and variable reach was significantly smaller than the difference between the constricted and variable reach conditions, which indicates that participants were estimating liberally rather than conservatively in the variable condition. Whereas in the current study, the difference between unconstricted and variable reach conditions did not significantly differ from the difference between the constricted and variable reach conditions, suggesting that a moderate-sized action boundary was selected.

One possible reason as to why the current findings did not conform to those in virtual reality may be the context in which the actions were learned. Previous studies were conducted in virtual reality and the perceptual system may handle variability differently in a real-world setting. Possibly, individuals are less conservative in virtual environments, because they are aware that the environment is not real. Hence, they might glean that they would not suffer the same consequences for failing as they would in the real world. However, this explanation is unlikely due to there being no real consequences for failing to successfully reach either environments. Another explanation for the differences could be that perceived distances are compressed in virtual environments in comparison to the real world (Loomis & Knapp, 2003). However, previous research has shown that the presence of a fully animated avatar nearly eliminates distance compression in virtual reality (Mohler et al., 2008). Moreover, if perceived distances were compressed in the virtual environment, the compression would apply to all calibration distances in all three conditions, hence this compression is unlikely to account for differences between the real and virtual environments.

Another reason as to why we found slightly different results here could be the way by which perceptual-motor variability was introduced. In the current study, we used a modified orthopedic elbow brace with the intention to simulate injury to the upper limb by restricting elbow extension. In the virtual study by Lin et al. (2020), we modified the length of the arm itself, which left the range of motion intact and arm movements in their natural state. In the case of this experiment, we restricted and modified the natural arm movement. Restricting elbow range of motion resembles the movement of the arm in a state of injury. Consider that much of the physical therapy following a serious injury to the arm, for example, bone breakage, involves gradually stretching the arm overtime to recover its range of motion. Perhaps, here, the perceptual system was treating the reduction in the range of motion as if it were a real injury. Hence, by selecting a less liberal action boundary the perceptual system may have been trying to maximize the probability of success while minimizing the probability of exacerbating a potential injury.

Therefore, the current findings could be a reflection of the differences in the methods used to manipulate reaching ability. Different manifestations of motor variability in the same actions produced different patterns of results. For instance, if perceptual-motor variability is introduced by inducing tremors or involuntary muscle contraction in arm muscles, while the arm’s length and ROM remain unchanged, the individual’s reaching ability will differ from one moment to the next due to inconsistent muscle contractions. Therefore, how the perceptual system selects an action boundary may be different in this situation. Consider, for example, the reaching ability variance that occurs in individuals with Parkinson’s and stroke patients, whose perceptual-motor feedback for reaching is constantly in flux due to abnormalities of neural and muscular activation (Mazzoni et al., 2012). Therefore, understanding the influence of perceptual-motor variability and the way in which it is manifested differently within a given action will provide valuable insights. Future research could explore these factors further by examining whether different strategies are employed for different manipulations.

In summary, the current study demonstrated that the manipulation of elbow range of motion can influence the perception of action boundaries in the real world. Our findings also show that when anticipating our reaching capability in the event of perceptual-motor variability in a real-world setting, individuals were not as liberal with their reachability estimates as they were in virtual reality. However, other factors such as the context, methodology, as well as the way in which variability is introduced to perceptual-motor feedback specifying one’s action boundary may also influence the size of the action boundary selected.

## Open Practices Statement

This experiment was not preregistered. The data that support the findings of this study will be made freely available in a repository after publication.
